# The golgin family exhibits a propensity to form condensates in living cells

**DOI:** 10.1002/1873-3468.13884

**Published:** 2020-08-02

**Authors:** Pascal Ziltener, Aleksander A. Rebane, Morven Graham, Andreas M. Ernst, James E. Rothman

**Affiliations:** ^1^ Department of Cell Biology Yale School of Medicine New Haven CT USA

## Abstract

The Golgi is surrounded by a ribosome‐excluding matrix. Recently, we reported that the cis‐Golgi‐localized golgin GM130 can phase‐separate to form dynamic, liquid‐like condensates *in vitro* and *in vivo*. Here, we show that the overexpression of each of the remaining cis (golgin160, GMAP210)‐ and trans (golgin97, golgin245, GCC88, GCC185)‐golgins results in novel protein condensates. Focused ion beam scanning electron microscopy (FIB‐SEM) images of GM130 condensates reveal a complex internal organization with branching aqueous channels. Pairs of golgins overexpressed in the same cell form distinct juxtaposed condensates. These findings support the hypothesis that, in addition to their established roles as vesicle tethers, phase separation may be a common feature of the golgin family that contributes to Golgi organization.

## Graphical abstract

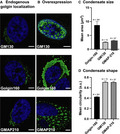

## Abbreviations

FIB‐SEM, focused ion beam scanning electron microscopy

ROI, regions of interest

In higher eukaryotes, the Golgi apparatus is an organelle characterized by a stack of disk‐like membrane compartments that are polarized from cis to trans, and plays a vital role as a sorting hub, where protein arriving from the ER is modified in a stepwise fashion and directed to intracellular and extracellular targets [1, 2]. While it is clear that the Golgi can be dynamically assembled and disassembled, as for instance during mitosis [3], the mechanisms by which this is achieved are incompletely understood. The interphase Golgi is polarized from cis to trans. For example, different glycosyltransferases are concentrated in successive cisternae, and asymmetric distributions of SNAREs and golgins are thought to enable the correct targeting of vesicle carriers within the stack [4, 5].

Golgins are long peripheral or membrane‐integral coiled‐coil domain‐containing proteins that exhibit selectivity toward their respective carrier subtypes, which has been demonstrated by experiments showing a selective accumulation of carriers following the artificial relocation of particular golgins to mitochondria [5]. The evidence thus supports a model in which golgins contribute to Golgi polarization by selectively binding distinct Rab GTPase isoforms found on different vesicle carriers. This interaction is thought to trigger the bending or collapse of the golgin [6, 7], which brings v‐SNAREs present on the carrier in proximity with complementary t‐SNAREs on the Golgi membrane, thus enabling a selective delivery of cargo to the Golgi.

In addition to their role in intra‐Golgi transport, golgins and other cytoplasmic matrix proteins have been proposed to laterally link individual ministacks into the continuous perinuclear Golgi ribbon [8]. There have been previous if indirect indications that golgins can preferentially self‐associate. For example, upon Golgi disassembly using brefeldin A, the golgins largely accumulate in cytoplasmic remnants separate from most of the pre‐existing Golgi membranes, whereas most of the organelle’s membranes including its integral proteins relocate to the ER by membrane fusion. And upon washout of brefeldin A, blockade of exit from the ER via microinjection of a Sar1 dominant‐negative mutant does not prevent golgins from reassembling the Golgi ribbon, while oligosaccharide‐modifying enzymes are retained in the ER [8]. This evidence suggests that golgins and or other matrix proteins could be involved in generating the higher‐order architecture of the Golgi by a mechanism that is independent of the bulk of other Golgi proteins [8]. Further, it has been shown that this relocation and assembly of the Golgi is dependent on the interaction of golgin160 with the minus‐end‐directed motor dynein, also implicating a specific golgin in the reassembly process [9]. We recently reported that the golgin GM130 phase separates into liquid‐like condensates both *in vitro* and *in vivo* [10], directly supporting the view that golgins can self‐assemble.

Here, employing overexpression of recombinant golgins combined with fluorescence and electron microscopy, we report that all seven peripherally anchored golgins in the cis‐ and trans‐Golgi (Fig. S1) exhibit a propensity to assemble into condensates within cells. In addition to their classical function as cognate vesicle tethers, we thus suggest [11] that golgins can also self‐assemble to form a dynamic scaffold or cocoon around the Golgi that could help shape, stabilize, or possibly even template the Golgi stack.

## Materials and methods

### Cloning of pCMV‐SNAP‐golgin160

The golgin160 gene was PCR‐amplified from plasmid OriGene RC221387 using forward primer CGCGTTTAAACTCGAGATGGACGGCGCGTCGGCC and reverse primer CAAATGATATCCTGGATTACAAGGATGACGACGATAAGCTCGAGGTTAATTAAT. The amplification product was purified using the QIAquick Gel Extraction Kit (Qiagen, 28704, Hilden, Germany) and subcloned into an Xho1‐digested (NEB, R0146S) pSNAPf mammalian expression vector (NEB N9183S) using In‐Fusion Cloning (Takara Bio, 638910, Kusatsu, Japan).

### Transfection, immunolabeling, and imaging

HeLa cells (ATCC CCL‐2) were grown at 37˚C and 5% CO_2_ in DMEM (Gibco, Grand Island, NY, USA) supplemented with 10% FBS (Gibco). 10^6^ cells were electroporated with 4 µg GM130 (OriGene [Rockville, MD, USA] RC209641), 4 µg golgin160 (OriGene RC221387), 4 µg golgin160‐GFP (OriGene RG221387), 8 µg GMAP210 (OriGene RC220674), 8 µg GMAP210‐GFP (OriGene RG220674), 8 µg golgin97 (OriGene RC206064), 8 µg golgin245 (OriGene RC230747), 8 µg GCC88 (OriGene RC204339), or 8 µg GCC185 (OriGene RC219795), using Nucleofector Kit R (Lonza #VVCA‐1001) and program I‐13 on a Nucleofector 2b device (Lonza AAB‐1001). Cells were grown on 12‐mm glass coverslips (Electron Microscopy Sciences 72230‐01,, Hatfield, PA, USA) coated with fibronectin (Millipore [Darmstadt, Germany] FC010) for fixation and immunostaining, or on 35‐mm glass‐bottom petri dishes (MatTek P35GC‐1.5‐14‐C) for live imaging.

Cells were fixed in 4% PFA (PBS) for 15 min, permeabilized (0.3% IGEPAL CA‐630, 0.05% Triton X‐100, 0.1% BSA), and blocked (5% goat serum, 0.05% IGEPAL CA‐630, 0.05% Triton X‐100) for an hour at RT. Primary antibodies were applied 1 hour at RT in blocking buffer, washed (0.05% IGEPAL CA‐630, 0.05% Triton X‐100, 0.2% BSA), followed by 1‐hour staining with secondary antibodies in blocking buffer at RT. Cells were then washed and mounted on glass slides with ProLong Gold Antifade Reagent with DAPI (Invitrogen [Carlsbad, CA, USA] P36931).

Live cells were imaged in DMEM without phenol red (Gibco 21063‐029). Cells were imaged on a ZEISS LSM 880 Airyscan confocal microscope.

### Data analysis

The confocal immunofluorescence images of cells overexpressing the various golgins were analyzed for condensate size and shape using ImageJ (29187165). The images were loaded into ImageJ and converted into binary masks using the ‘Threshold’ tool. Next, individual condensates were identified in the mask using the ‘Analyze Particles’ tool, and their shape and size parameters, such as condensate area and circularity, were determined. Statistical analysis was performed using graphpad prism 8 (GraphPad Software, La Jolla, CA, USA) for the mean and standard error of the mean (SEM) of the condensate area and circularity.

### Focused ion beam scanning electron microscopy

HeLa cells were transfected as described above with GFP‐GM130 [10] for 20 hours or golgin160‐GFP (OriGene RG221387) for 10 hours in T75 flasks, harvested with 0.05% trypsin (Thermo Fisher Scientific [Waltham, MA, USA] 25300054) for 4 min, and fixed for 10 min with 4% PFA (PBS). GFP^+^ cells were sorted using a BD FACSAria II and fixed in 2.5% glutaraldehyde and 2% paraformaldehyde in 0.1M sodium cacodylate buffer pH 7.4 containing 2% sucrose for 1 hour, then rinsed in buffer, and replaced with 0.1% tannic acid in buffer for another hour. Tissue samples were trimmed and postfixed in 1% osmium tetroxide and 1.5% potassium ferrocyanide in buffer for 1 hour. The samples were rinsed in sodium cacodylate and distilled water and en‐bloc stained in aqueous 0.5% uranyl acetate overnight. This was followed by further rinsing in distilled water and then placed in 60˚C lead aspartate for 1 hour. After 1‐hour rinsing in distilled water, the samples were dehydrated through an ethanol series to 100% and were infiltrated with Embed 812 (Electron Microscopy Sciences) resin, placed in silicone molds, and baked at 60°C for at least 24 hours.

The resin block was trimmed to rough area of interest and the surface cleanly cut. The entire pyramid was carefully removed with a fine blade and mounted on an aluminum stub using conductive carbon adhesive and silver paint (Electron Microscopy Sciences) and then sputtered with approximately 20 nm Pt/Pd (80/20) using a Cressington HR sputter coating equipment (Ted Pella, Inc., Redding, CA, USA) to reduce charging effects.

A dual‐beam focused ion beam scanning electron microscopy (FIB‐SEM) (ZEISS Crossbeam 550) using a Gallium ion source was used to mill, and SE2 secondary electron detector was used to image the samples. SmartSEM (ZEISS, Jenna Germany) was used to set up initial parameters and to find the regions of interest (ROI) by SEM images at 10 kV. 50 µm width X 30 µm height X actual depth was 30 µm with 7 nm/pixel and 7 nm per slice. A platinum protective layer was deposited at the ROI with the FIB (30 kV, 50 pA) to protect the structure and reduce charging. Carbon deposits mill, and highlight was done at 30 kV and 3 nA. A course trench milled 30 kv 30 nA followed by fine milling at 30 kV 3 nA. For final acquisition of a cuboid, the area of interest was milled at 30 kV and 300 pA. After milling each slice, an image was taken by detecting backscattered electrons of a primary electron beam (acceleration voltage of 1.5 kV, imaging current of 2 nA, and aperture diameter of 100 µm) with a pixel dwell time of 2 µs. Atlas 5 (ZEISS) was used for preliminary SEM stack alignment, and FIB‐SEM image stacks were saved as tif and MRC files. The images were imported into Dragonfly software (ORS, Montreal Canada) for further alignment and segmentation.

### Antibodies

Commercial antibodies used were GM130 (BD Biosciences 610823), golgin160 (Sigma [St. Louis, MO, USA] HPA039809), GMAP210 (Sigma HPA070684), golgin97 (Thermo Fisher Scientific A‐21270), golgin245 (BD Biosciences [San Jose, CA, USA] 611281), GCC88 (Sigma HPA019369), GCC185 (Sigma HPA035849), and Flag (Sigma F1804, F7425). All antibodies were validated for their specificity by confocal microscopy using the expression of constructs of the golgin in question (listed above) fused to a FLAG tag.

## Results and Discussion

### Condensates formed from cis‐Golgi‐localized golgins GM130, Golgin160, and GMAP210

**Fig. 1:**
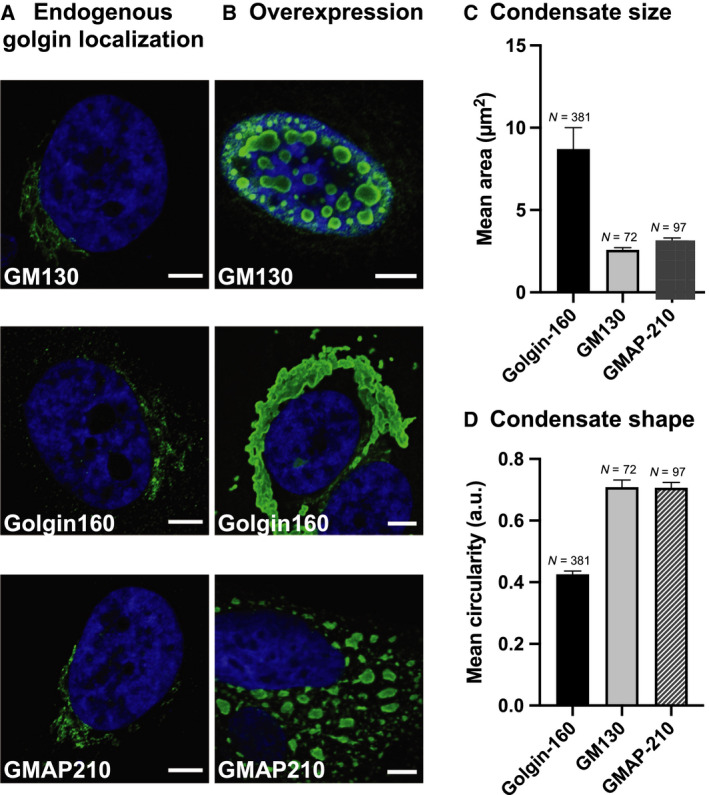
Overexpressed cis‐golgins form condensates. (A) HeLa cells were fixed and immunostained for endogenous golgins or (B) HeLa cells were transfected with GM130‐FLAG/Myc, golgin160‐FLAG/Myc, or GMAP210‐FLAG/Myc, respectively, fixed 6‐24 h post‐transfection, and immunostained for FLAG (exogenous golgin). Secondary antibody conjugated to Alexa Fluor 488 was used in (A) and (B). Overexpressed GM130 forms condensates in the nucleus, while golgin160 and GMAP210 form condensates in the cytoplasm. (C) Mean condensate area and (D) mean condensate circularity, where N denotes the number of condensates analyzed and error bars denote the standard error of the mean (SEM). Only condensates with area >1.5 µm^2^ were considered in this analysis, in order to retain an accurate measure for circularity. Scale bars: 5 µm

Building on our finding that the purified cis‐Golgi‐localized golgin GM130 can phase‐separate into liquid‐like condensates, we set out to test whether the other two golgins in the cis‐Golgi, golgin160 and GMAP210, showed a similar propensity to form condensates. Both golgin160 and GMAP210 are rod‐like coiled‐coil domain‐containing proteins that are peripherally anchored to the cis‐Golgi membrane by the small GTPase ARF1 ([12], Fig. S1). At endogenous levels, as judged from immunolabeling and confocal microscopy, the cis‐face‐localized golgins exhibit a largely perinuclear, ribbon‐like localization pattern, with golgin160 additionally localizing to cytoplasmic puncta (Fig. 1A). Upon overexpression of Myc/FLAG‐tagged GM130 for 6‐24 h, the protein accumulated in spherical droplets in the nucleus as we previously described for GFP‐GM130 [10]. These condensates grow to 2.6 ± 0.1 µm^2^ foci that appear to be able to fuse and relax back into spherical shapes (Fig. 1B, top panel). This is likely due to the fact that the overexpressed GM130 overwhelms the endogenous pool of p115 which normally masks the amino‐terminal nuclear localization signal (NLS) and prevents import of GM130 into the nucleus [13]. In contrast to GM130, both golgin160 and GMAP210 accumulated in the cytoplasm after 6 h of overexpression. Golgin160 condensates were larger in size (mean area of 9 ± 1 µm^2^, Fig. 1C) and more irregularly shaped (Fig. 1D) compared with GM130 and GMAP210, forming extensive networks that wrap around the nucleus (Fig. 1B, middle panel). Interestingly, overexpression of GMAP210 resulted in the formation of rather spherical condensates (Fig. 1D) with mean area of 3.2 ± 0.2 µm^2^ (Fig. 1C), which were distributed throughout the cytoplasm (Fig. 1B, bottom panel). As suggested by the shape, FRAP experiments show partial fluidity of GMAP210 condensates, while the irregularly shaped golgin160 condensates appear to have hardened (Fig. S3).

Our results prompt the view that the cis‐Golgi‐localized golgins share a propensity to condense into structures that in most cases are reminiscent of dynamic, liquid‐like assemblies.

### Condensates formed from trans‐Golgi/TGN‐localized golgins golgin97, golgin245, GCC88, and GCC185

**Fig. 2:**
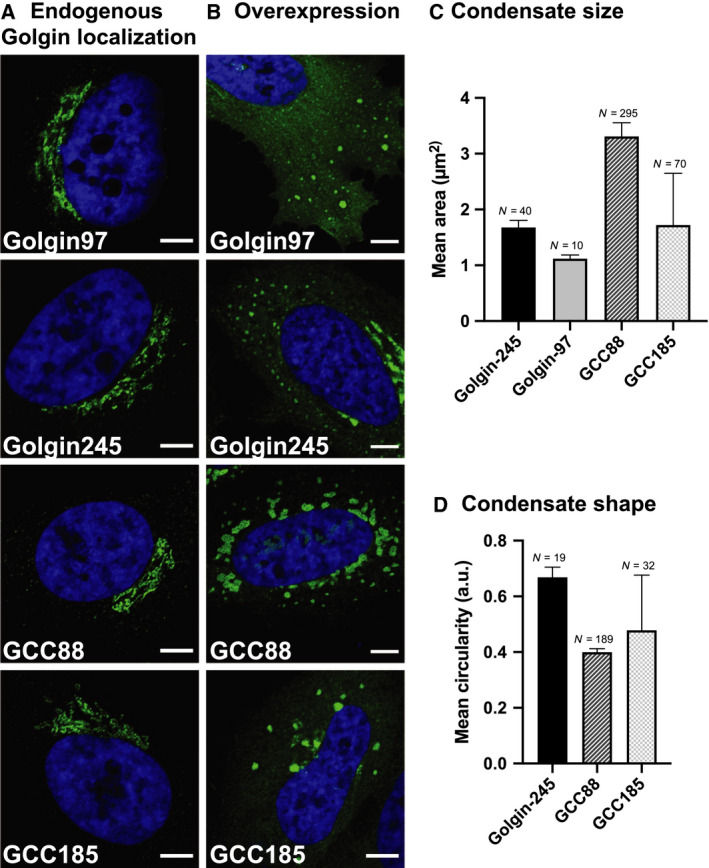
Overexpressed trans‐golgins form condensates. (A) HeLa cells were fixed and immunostained for endogenous golgins or (B) HeLa cells were transfected with golgin97‐FLAG/Myc, golgin245‐FLAG/Myc, GCC88‐FLAG/Myc, or GCC185‐FLAG/Myc, respectively, fixed 6 h post‐transfection and immunostained for FLAG (exogenous golgin). Secondary antibody conjugated to Alexa Fluor 488 was used in (A) and (B). Each overexpressed golgin formed characteristic condensates. (C) Mean condensate area and (D) mean condensate circularity, where N denotes the number of condensates analyzed and error bars denote the standard error of the mean (SEM). The mean area (and SEM) was calculated based on condensates with area >0.8 µm^2^, thereby excluding contributions from random fluctuations in protein concentration or fluorescence signal. The mean circularity (and SEM) was calculated by including condensates with area >1.5 µm^2^ because circularity became ill‐defined for smaller particles. Golgin97 was excluded for the circularity analysis because too few condensates satisfied these criteria for accurate statistics. Scale bars: 5 µm.

Next, we set out to test whether the trans‐Golgi/TGN‐localized golgins golgin97, golgin245, GCC88, and GCC185 could condense into large foci as observed for the cis‐golgins. These golgins exhibit GRIP domains [14] that anchor these vesicle tethers via the small GTPase ARL1 to the trans‐Golgi/TGN. At endogenous levels, these golgins exhibited a ribbon‐like, perinuclear localization (Fig. 2A).

Strikingly, as observed for the overexpressed cis‐Golgi‐localized golgins, the overexpression of golgin97, golgin245, GCC88, and GCC185 also resulted in the formation of condensates 6 h post‐transfection (Fig. 2B). While golgin97 and golgin245 formed spherical condensates, which accumulated in the cell periphery and appeared smaller than GMAP210 condensates (mean area of 1.1 ± 0.1 µm^2^ for golgin97 and 1.6 ± 0.1 µm^2^ for golgin245), those formed by GCC185 were distributed throughout the cytoplasm and appeared larger, with mean area 1.7 ± 0.1 µm^2^ (Fig. 2B–D). GCC88 condensates were slightly larger (mean area 3.3 ± 0.2 µm^2^) and exhibited an irregular morphology reminiscent of the golgin160 condensates (Fig. 2B–D). As with golgin160, GCC88 condensates appear to have hardened as determined by FRAP, while the much more circlular golgin97 condensates appear to be partially fluid (Fig. S3). A previous report also described electron‐dense structures formed by GCC88 upon overexpression, though it did not link the structures to the concept of phase separation, which had not yet been forwarded [15]. As described above for the cis‐face of the Golgi, condensation of the major trans‐Golgi/TGN golgins could provide a mechanism to specify the trans‐face of the stack, first by self‐assembling into distinct domains, and then by populating these by the content of their cognate vesicles in their roles as tethers [11, 16].

In addition to the cis‐ and trans‐golgins, there are also medial golgins, such as giantin, which are anchored via carboxyterminal transmembrane domains and localize to the rims of the Golgi stack [12]. Given that they share a similar anatomy to the cis‐ and trans‐golgins, it is reasonable to expect that these medial golgins might also be able to condense into foci, though we did not test this in the present study.

### Fates of cis‐golgin condensates over time

**Fig. 3:**
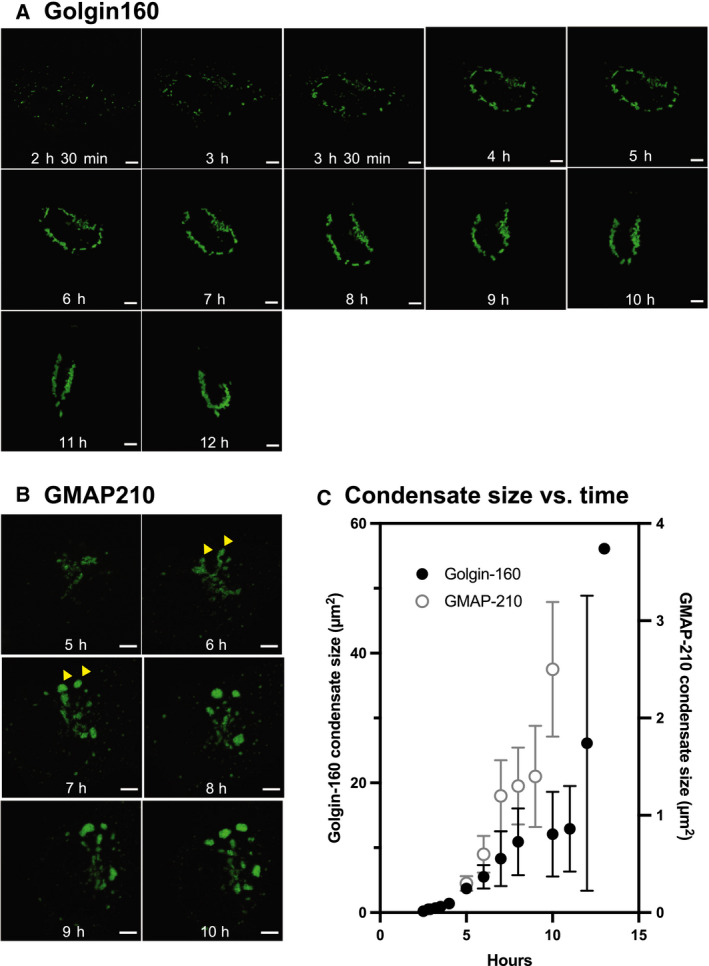
Time‐lapse microscopy of condensate formation in live cells. Live‐cell confocal microscopy of a single cell transfected with (A) golgin160‐GFP or (B) GMAP210‐GFP tracked over time. (C) Mean condensate area vs. time, as determined from the images shown in (A) and (B). Golgin160 is plotted as filled black circles on the left ordinate; GMAP210 is plotted as open gray circles on the right ordinate. Scale bars: 5 µm.

We previously showed that GM130 condensates form and fuse over a period of hours [10]. Live imaging tracking a single cell over time revealed that golgin160 condensates first appear as small condensates surrounding the nucleus at 2.5 h post‐transfection (Fig. 3A), and grow by coalescing into larger structures over a period of around 10 hours (Fig. 3C, solid circles) that encircle the nucleus (Fig. 1B). This convergence of small condensates into one structure is likely driven by dynein movement to minus‐end microtubules, as it has been shown that golgin160 binds directly to dynein and that this interaction is required for pericentrosomal Golgi localization during interphase [9].

GMAP210 condensates also first appear encircling the nucleus, primarily in the Golgi region (Fig. 3B, 5h). Strikingly, over the course of 2 h, increasingly abundant GMAP210 then concentrates at the rims of the ribbon (Fig. 3B, 6h arrowhead), apparently leading to the detachment of spherical condensates (Fig. 3B, 7h arrowhead). Over the course of a few hours, multiple large spherical condensates grew in size (Fig. 3C), now found throughout the cytoplasm (Fig. 1B; Fig. 3B).

As observed for all golgins in this study, the presence of smooth borders around the condensates as well as their dynamics indirectly supports that these condensates may exhibit liquid‐like properties.

### Internal structure of GM130 and golgin160 condensates

The propensity of golgins to undergo a liquid–liquid phase separation is unexpected, since these proteins do not have extensive disordered domains ([17], Fig. S1), which have been shown to drive the phase separation of an increasing list of proteins described in the literature (reviewed in [18]). Rather, their condensation seems to occur due to interactions among their coiled‐coil domains [10] which are likely to be numerous but weak. To more closely analyze the nature of the golgin condensates formed by overexpression, we employed FACS sorting of Hela cells transiently transfected with GFP‐GM130 and golgin160‐GFP to select the most abundantly expressing cells for focused ion beam electron microscopy (FIB‐SEM).

**Fig. 4:**
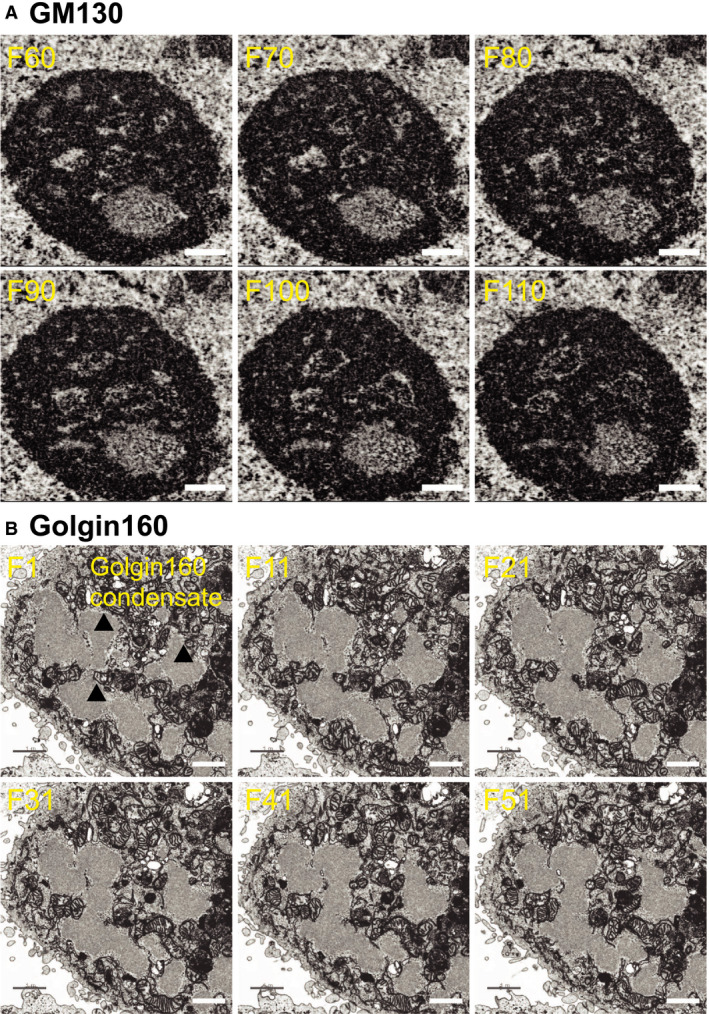
FIB‐SEM of GM130 and golgin160 condensates. HeLa cells were transfected with (A) GFP‐GM130 for 20 h or (B) golgin160‐GFP for 10 h, enriched for GFP^+^ cells using a BD FACSAria II cell sorter, and visualized using FIB‐SEM. GM130 condensate in (A) is seen as a proteinaceous sphere (black) riddled with aqueous channels (white). Golgin160 condensates in (B) are indicated by arrowheads. The frame of the FIB‐SEM tomogram is indicated in the top left corner of each panel. Scale bars are 500 nm for (A) and 1 µm for (B).

These FIB‐SEM images of GM130 nuclear condensates clearly reveal a complex internal organization that is characterized by nonisotropic distribution. These condensates are in fact sponge‐like structures consisting of a continuous matrix of protein filled with numerous branching and anastomosing aqueous channels, not unlike that of a gel used for size fractionation of proteins (Fig. 4A). The thickness of the continuous protein matrix, as measured between adjacent voids, is ~ 86 ± 8 nm, consistent with models in which GM130 is associated laterally in layers 1‐2 molecules thick arranged locally in mainly lamellar fashion. There is no evidence of membranes within the condensates (Fig. 4A).

Similarly, cytoplasmic condensates formed by the overexpression of golgin160 do not contain membranes (Fig. 4B). The golgin160 condensates also show evidence of internal structure, yet the condensates appear more protein‐rich and protein‐dense than the GM130 condensates and lack prominent aqueous channels (Fig. 4B). Large golgin160 condensates that have presumably coalesced together also have a smooth border, which is consistent with a liquid‐like nature of the condensates.

### Different golgins locate to distinct condensates

**Fig. 5:**
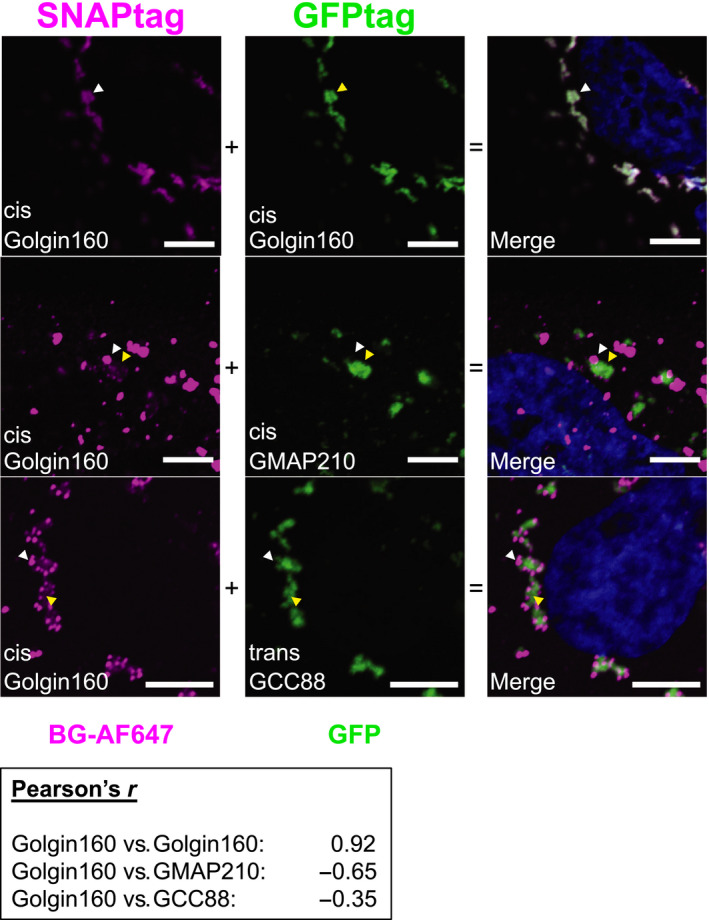
Separate but associated condensates of overexpressed pairs of golgins. HeLa cells were transfected with 2 µg SNAP‐golgin160 and 2µg golgin160‐GFP, or 2 µg SNAP‐golgin160 and 8 µg GMAP210‐GFP, or 2 µg SNAP‐golgin160 and 8 µg GCC88‐GFP as indicated and fixed 6 h post‐transfection. Golgin condensates were visualized by direct GFP fluorescence or by staining postfixation with BG‐AF647 using a confocal microscope. White and yellow arrowheads indicate SNAP or GFP‐fused golgin‐dominated condensates, respectively. Scale bars: 5 µm

To investigate the specificity of the condensate interactions, we overexpressed pairs of golgins within the same cell fused to SNAPtag and GFP, respectively. As a control to rule out technical artifacts (Fig. 5, top panel), the same cis‐golgin (golgin160) bearing different tags (SNAP and GFP) was overexpressed in the same cell. This resulted in homogenous mixing of the two species (Pearson’s score of 0.92). In contrast, the two distinct cis‐golgins, SNAP‐golgin160 and GMAP210‐GFP located to mainly distinct condensates in the same cell (Fig. 5 middle panel). Distinct condensates also resulted when a cis‐Golgin (SNAP‐golgin160) and a trans‐golgin (GCC88‐GFP) were co‐expressed (Fig. 5 bottom panel). In both of these cases, the Pearson score was negative. The simplest interpretation of these results is that the different golgins form separate condensates, perhaps reflecting their differential localizations within the native Golgi complex. However, we currently cannot rule out the alternative that the golgins mix initially in a liquid state, and then separate from each other as they harden with time.

Another interesting feature is that the distinct condensates are almost always found in close proximity, indeed in contact at optical resolution (indicated by white and yellow arrowheads, Fig. 5 middle and bottom panels). If this were due to direct adhesion of the condensates, we could conclude that the golgins, regardless of cis‐ or trans‐identity, have an affinity for each other but a larger affinity to associate with self, as predicted by a self‐assembly model for the Golgi [11]. However, at present resolution we cannot exclude that the condensates adhere independently to a common framework rather than directly to each other. Future *in vitro* studies will be needed to differentiate among these possibilities.

In summary, our data suggest that the propensity to form condensates in living cells is a common feature among proteins of the golgin family. We have shown from rigorous investigation of the physical–chemical properties of the isolated cis‐golgin GM130 that this golgin undergoes liquid–liquid phase separation at concentrations that are reached within the Golgi [10]. Analogous studies of the other golgins will be required to determine whether the biological condensates form similarly. The molecular features that cause golgins to phase‐separate or form condensates have yet to be determined. However, the internal organization of the GM130 condensates revealed by electron microscopy suggests that GM130 assemble locally into flexible sheets 1‐2 molecules thick that branch and anastomose to create aqueous channels, most easily explained by lateral association of these flexible, rod‐like molecules. Since all golgins are integral or peripheral membrane proteins, it is likely that their orientation on Golgi membranes could influence their interactions and/or miscibility with each other. Golgins are thus ideally positioned to define the long observed zone of exclusion or cocoon that encases the Golgi and excludes ribosomes—a dynamic, self‐organizing, liquid‐like phase of golgins and matrix proteins.

## Author contributions

PZ and MG performed experiments. PZ, AAR, AME, and MG analyzed data and contributed to portions of the manuscript. PZ, AME, and JER designed research and wrote the manuscript.

## Supporting information


**Fig. S1.** Schematic of cis and trans‐golgins.
**Fig. S2.** FIB‐SEM tomograms of GM130 and golgin160 condensates.
**Fig. S3.** In vivo FRAP of golgin condensates.
