# Supplementary material for: The golgin family exhibits a propensity to form condensates in living cells
Source: FEBS Lett. 2020 Aug 2;594(19):3086–94. doi: 10.1002/1873-3468.13884 (PMC7589415; doi:10.1002/1873-3468.13884)
Supplement: Supplementary file 1 — Fig. S1. Schematic of cis and trans‐golgins. Fig. S2. FIB‐SEM tomograms of GM130 and golgin160 condensates. Fig. S3. In vivo FRAP of golgin condensates. [file FEB2-594-3086-s001.pptx]

## Slide 1
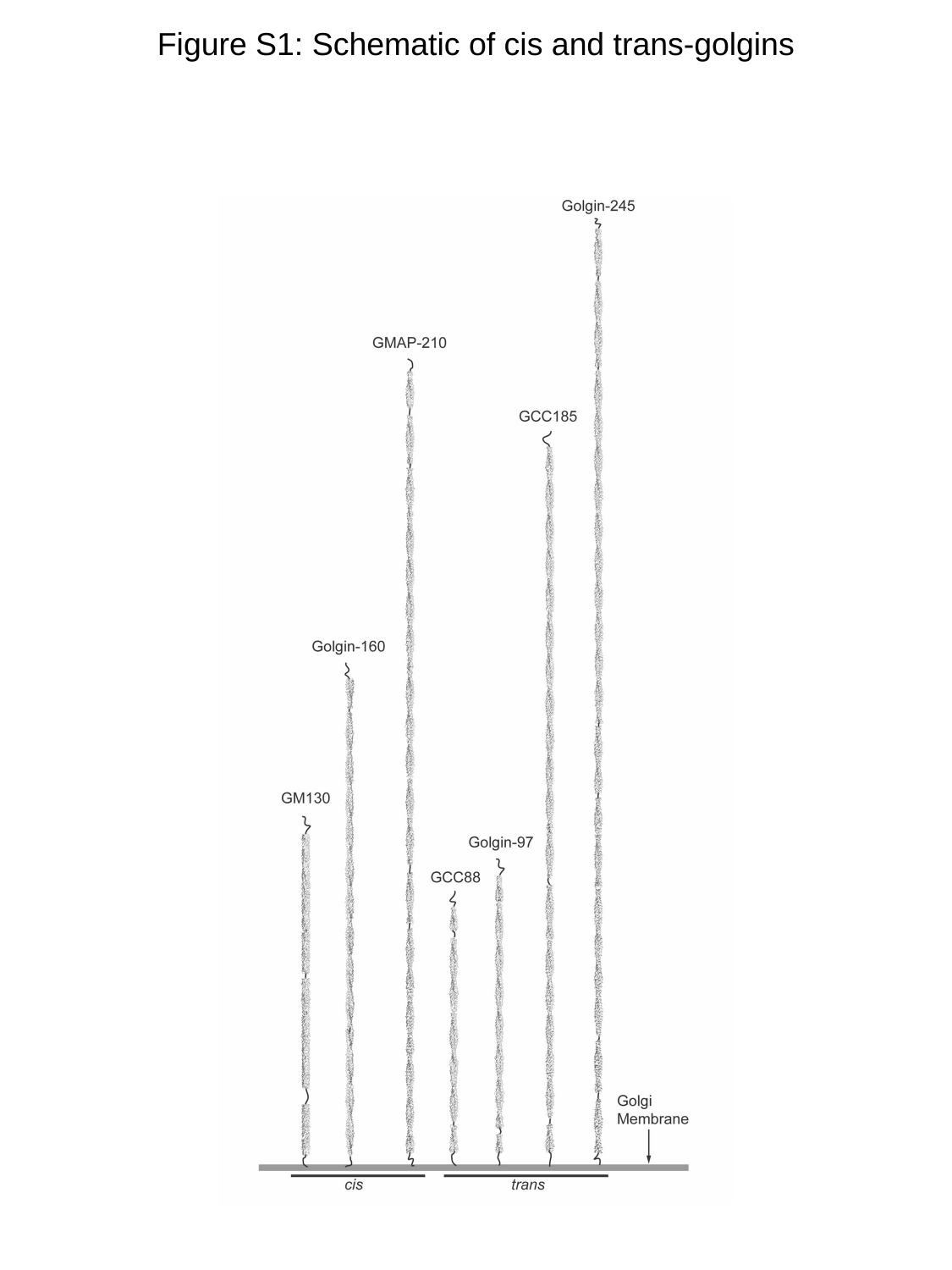

Figure S1: Schematic of cis and trans-golgins

## Slide 2
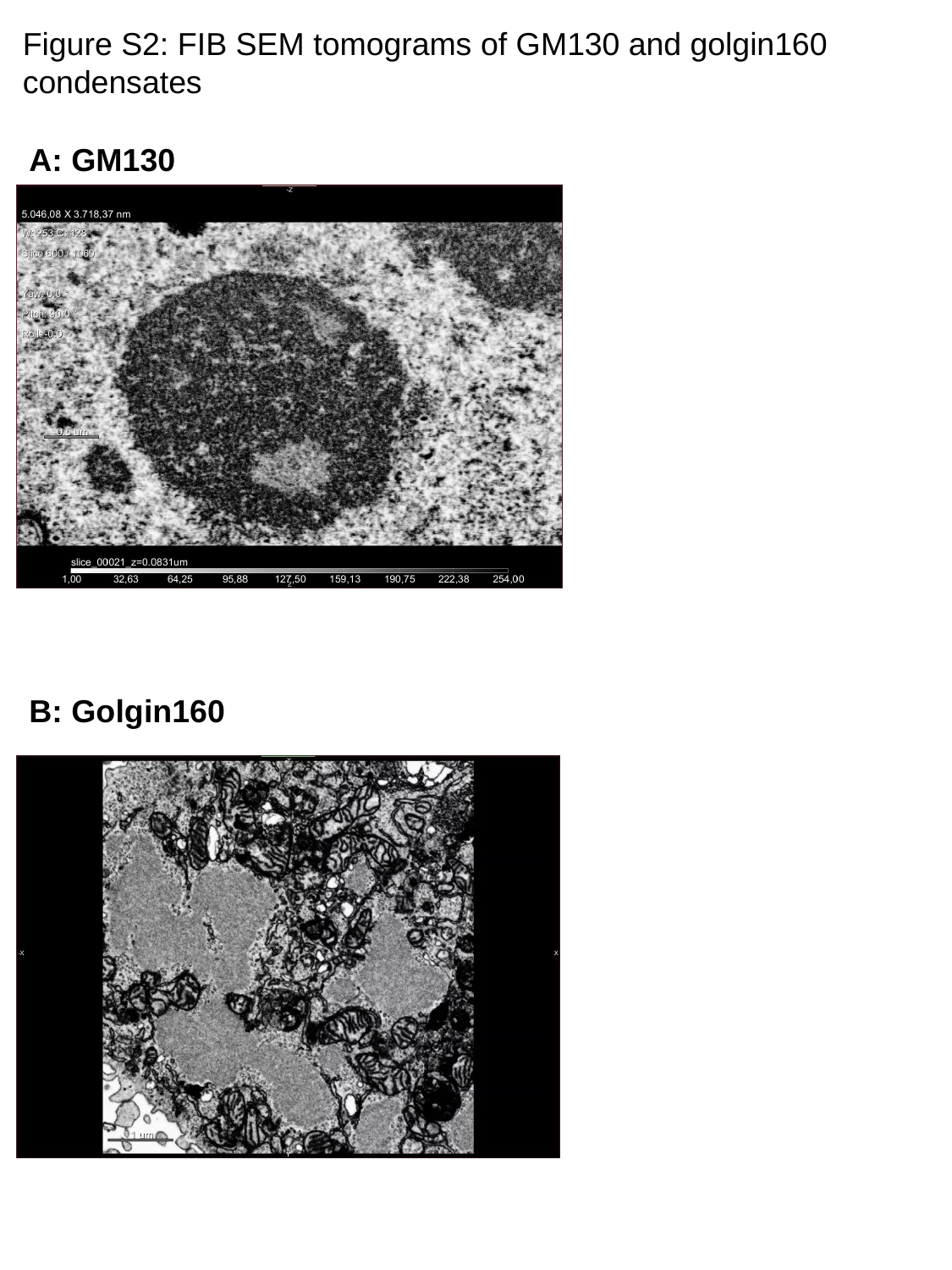

Figure S2: FIB SEM tomograms of GM130 and golgin160 condensates
A: GM130
B: Golgin160

## Slide 3
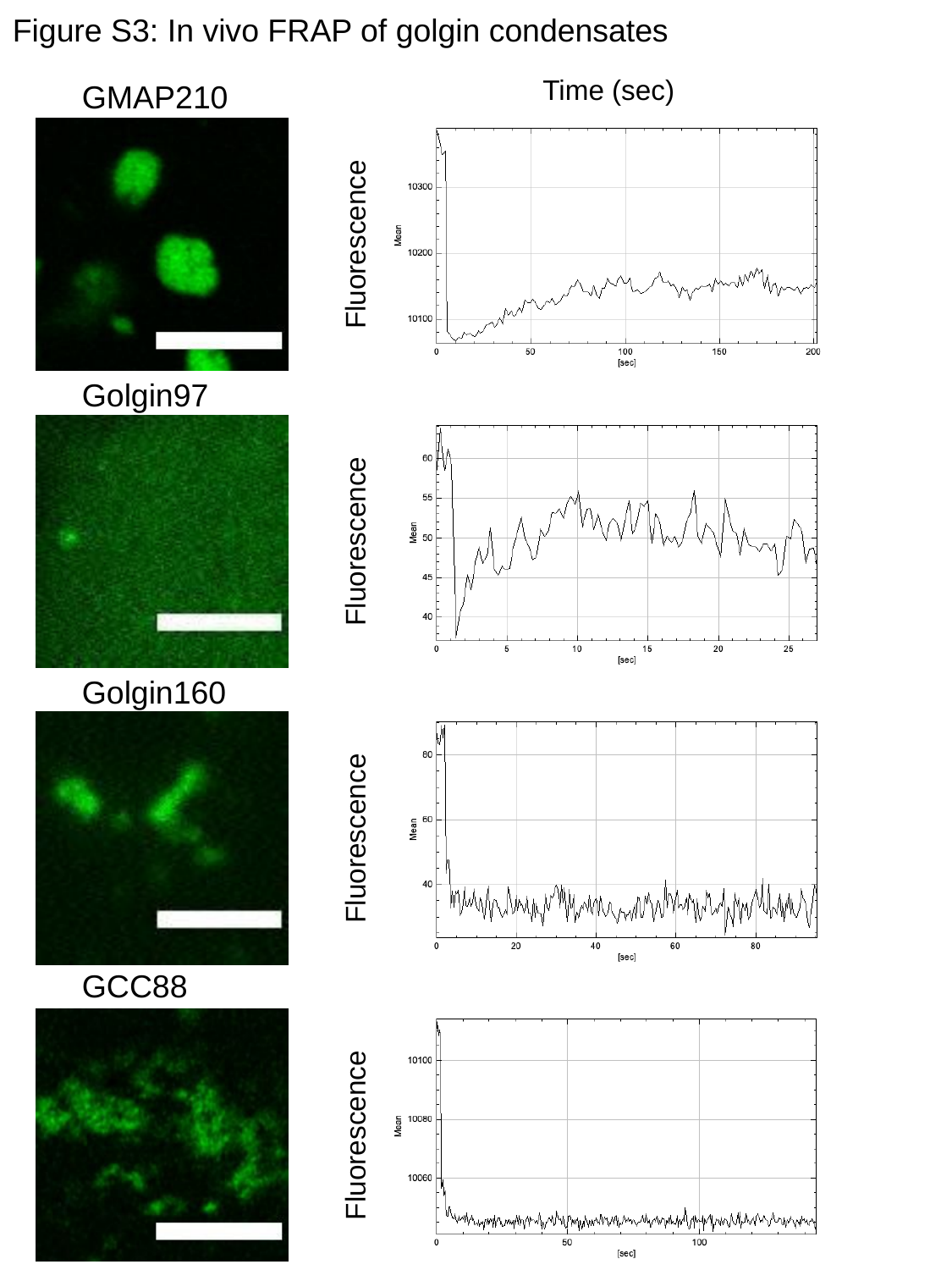

Figure S3: In vivo FRAP of golgin condensates
Time (sec)
GMAP210
Fluorescence
Golgin97
Fluorescence
Golgin160
Fluorescence
GCC88
Fluorescence
